# The prevalence of conduct disorders among young people in europe: A systematic review and meta-analysis

**DOI:** 10.1192/j.eurpsy.2021.1693

**Published:** 2021-08-13

**Authors:** R. Sacco, N. Camilleri, K. Umla-Runge

**Affiliations:** 1 Psychiatry, Cardiff University, Teesside University, Malta Mental Health Services, Attard, Malta; 2 Child And Young People’s Services, Malta Mental Health Services, Pieta, Malta; 3 Psychiatry, Cardiff University, Cardiff, United Kingdom

**Keywords:** Child, adolescent, Conduct, prevalence

## Abstract

**Introduction:**

This systematic review estimates the pooled prevalence (PP) of Conduct Disorder (CD) among 5-to-18-year-old YP living in Europe, based on prevalence rates established in the last five years (LFY).

**Objectives:**

Trends of prevalence rates across countries, gender and level of education were analysed. The random effects pooled prevalence rate (REPPR) for CD was calculated.

**Methods:**

A search strategy was conducted on three databases. Studies were also identified from reference lists and grey literature. Eligible studies were evaluated for reliability, validity and bias, and REPPRs were calculated.

**Results:**

The European REPPR for CD is calculated at 1.5% (Figure1). The REPPR among males is 1.8% whereas the rate among females is 1.0% (Figure2). The prevalence rate of CD in primary school children is 1.4 times lower than the prevalence of secondary school children.

**Figure:**
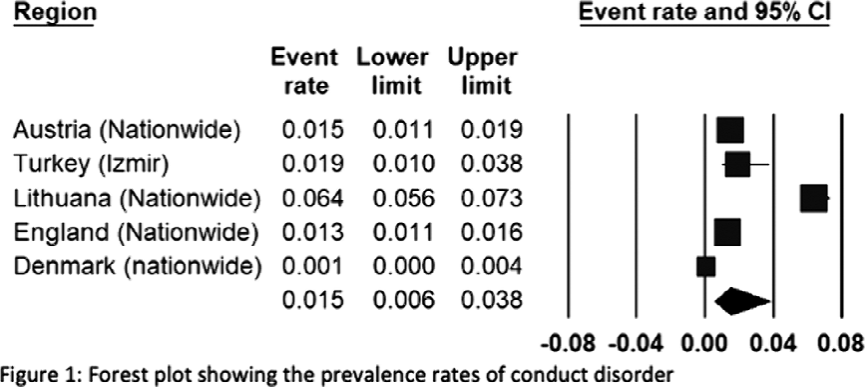

**Figure:**
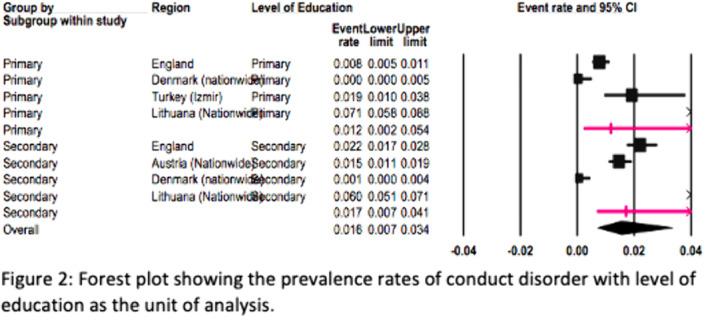

**Conclusions:**

Gender, culture and socioeconomic inequality may contribute towards diagnostic inequality and prevalence differences. It is recommended that these aspects are addressed, and routine screening and early intervention services are developed.

**Disclosure:**

No significant relationships.

